# Seeking care in the context of social health insurance in Kenya and Ghana

**DOI:** 10.1186/s12889-020-08742-1

**Published:** 2020-05-04

**Authors:** Lauren Suchman, Catherine Verde Hashim, Joseph Adu, Rita Mwachandi

**Affiliations:** 1 Institute for Global Health Sciences, University of California San Francisco, San Francisco, CA USA; 2 Marie Stopes International, London, UK; 3 Marie Stopes Ghana, Accra, Ghana; 4 Population Services Kenya, Nairobi, Kenya

**Keywords:** Social health insurance, Health-seeking behavior, Universal health coverage, Ghana, Kenya

## Abstract

**Background:**

Social Health Insurance (SHI) is widely used by countries attempting to move toward Universal Health Coverage (UHC). While evidence suggests that SHI is a promising strategy for achieving UHC, low-income countries often struggle to implement and sustain SHI systems. It is therefore important to understand how SHI enrollees use health insurance and how it affects their health-seeking behavior. This paper examines how SHI affects patient decision-making regarding when and where to seek care in Kenya and Ghana, two countries with established SHI systems in sub-Saharan Africa.

**Methods:**

This paper draws from two datasets collected under the African Health Markets for Equity (AHME) program. One dataset, collected in 2013 and 2017 as part of the AHME qualitative evaluation, consists of 106 semi-structured clinic exit interviews conducted with patients in Ghana and Kenya. This data was analyzed using an inductive, thematic approach. The second dataset was collected internally by the AHME partner organizations. It derives from a cross-sectional survey of social franchise clients at three social franchise networks supported by AHME. Data collection took place from February – May 2018 and in December 2018.

**Results:**

Many clients appreciated that insurance coverage made healthcare more affordable, reported seeking care more frequently when covered with SHI. Clients also noted that the coverage gave them access to a wider variety of providers, but rarely sought out SHI-accredited providers specifically. However, clients sometimes were charged for services that should have been covered by insurance. Due to a lack of understanding of SHI benefits, clients rarely knew they had been charged inappropriately.

**Conclusions:**

Clients and providers would benefit from education on what is included in the SHI package. Providers should be monitored and held accountable for charging clients inappropriately; in Ghana this should be accompanied by reforms to make government financing for SHI sustainable. Since clients valued provider proximity and both Kenya and Ghana have a dearth of providers in rural areas, both countries should incentivize providers to work in these areas and prioritize accrediting rural facilities into SHI schemes to increase accessibility and reach.

## Background

Universal financial protection and access to healthcare make up the backbone of the concept of universal health coverage (UHC) [1–3]. Social health insurance (SHI), which involves fund and risk pooling, is widely accepted as a key instrument for moving towards UHC [1, 4]. Several studies of SHI have shown that insurance programs can work in low-income areas and that members of an insurance scheme have a higher probability of using healthcare services than non-members [5, 6]. Some have found significant increases in uptake particularly by people living in poverty, women, and children, although proximity to a health facility also played a role in determining health-seeking behavior in this case [7]. Indeed, location is a common determinant of provider choice and frequency of visits [8–10].

Despite their promise, SHI schemes often have difficulties achieving UHC. While evidence indicates that the introduction of an SHI scheme can significantly increase equity in financing and access to healthcare services [6, 11], factors such as mistrust in the public health system [12], a lack of transparency in government [13], low re-enrollment rates [14], and a lack of sustainable funding [15] can hinder countries’ progress toward UHC. Consequently, some researchers suggest that policy and program design needs to be such that enrolment is effectively compulsory in practice [14] and SHI schemes should not only focus on the design of a viable SHI package, but should also involve stakeholder engagements and enhance transparency [13]. Yet, the question remains whether SHI schemes can continue to reasonably offer a large package of services to members within their limited risk pools without depleting funds [16].

Given that SHI has so much promise, but also faces so many obstacles to success, it is important to understand how SHI enrollees use health insurance and how it affects, or does not affect, their health-seeking behavior. However, there is a dearth of qualitative literature analyzing the complexities of patient experiences under SHI in low- and middle-income countries (LMICs) that might better inform policy implementation in these settings. This paper seeks to fill this gap by examining how SHI affects patient decision-making regarding when and where to seek care in Kenya and Ghana, two countries with established SHI systems in sub-Saharan Africa. Using client exit interviews, we examine whether patients covered by SHI felt that insurance coverage made healthcare more accessible and whether they reported seeking formal care more frequently than they would have without the coverage. We also analyze the extent to which provider choice was affected by providers’ SHI accreditation status.

### Literature review

A review of the literature indicates that having SHI coverage under the Ghana National Health Insurance Scheme (NHIS) has a significant positive impact on health service utilization [17–19]. In Ghana, the insured are significantly more likely to seek formal healthcare than those who are uninsured [20], and a comprehensive assessment of the health sector [6] concluded that when people living in poverty have insurance they are more likely to use a health facility than a poor individual who is uninsured. Further, some studies suggest that reasons for enrollment in the NHIS include perceived cost effectiveness and peace of mind [21, 22], and that financial protection is one of the main reasons for renewal [23].

Prior to the expansion of social health insurance in Kenya, the existing policy of user fees and other out-of-pocket payments (OOPs) led to massive decline in the utilization of health services [24, 25]. However, perhaps because much of Kenya’s population was effectively excluded from outpatient coverage under the National Hospital Insurance Fund (NHIF) until recently and because the NHIF has not been studied as extensively as the Ghana NHIS, it is unclear to what extent having NHIF coverage actually affects health-seeking behavior for non-specialized conditions [26–28]. This is a significant gap in the literature on the NHIF and healthcare in Kenya more broadly that this paper seeks to help close.

In both Ghana and Kenya, those who are wealthy and more highly educated are significantly more likely to enroll in and renew their membership in SHI schemes than their lower-income, less educated counterparts [29–31]. In Ghana, fully insured households are older, have a relatively higher number of household members with at least secondary education, and have more formal sector workers than those that are partially insured or uninsured. Partially insured households, on the other hand, have higher numbers of children and elderly members, while uninsured households have fewer elderly, formal sector workers, and members with secondary or higher education [32].

These demographics align with other findings that potential enrollees in Ghana were deterred from enrolling due to lack of affordability, being healthy, and lack of confidence in the scheme [23], as well as difficulties with the enrollment process [32]. Further, poor individuals in Ghana are less likely to seek healthcare, despite enrollment in NHIS [33]. Even after enrolling, studies have found that NHIS enrollees in Ghana are likely to drop out due to lack of affordability, perceived limited benefits, and poor service quality [29, 34]. A couple of studies also found evidence of adverse selection, suggesting that Ghanaians are more likely to renew their NHIS membership when sick, while allowing their enrollment to lapse during periods of good health [21, 35].

In both Kenya and Ghana the probability of having NHIF coverage increases with age, level of education, residence, and wealth status [36]. In addition, data from Kenya’s 2014 Demographic and Health Survey indicates that gender is associated with having health insurance, as men are slightly more likely to have coverage than are women [37]. In Kenya, a complex enrollment process, unclear communication regarding benefits from NHIF, and lack of affordability serve as the main deterrents to enrollment [38, 39].

However, even in cases where clients successfully enroll in the NHIF and NHIS, and maintain their enrollment, they often are deterred from using it by informal payments requested by providers or poor treatment when they try to pay with NHI as opposed to paying out of pocket. In both countries, patients attempting to pay with SHI coverage were often levied out-of-pocket fees, suggesting there is variance between the actual SHI package and the benefits received by patients on the ground [19, 38]. Further, patients in Kenya reported being discriminated against when they used their NHIF cards [38], while patients in Ghana who suspected they were being charged incorrectly did not mention this to providers for fear of negative treatment [40]. Thus, while the literature seems to suggest that SHI enrollment status holds promise for encouraging enrollees to seek healthcare more often, given the constraints both current and potential enrollees face in Kenya and Ghana it is unclear how much having SHI coverage affects more minute aspects of care-seeking behavior, such as provider choice. Further, much of this literature uses quantitative surveys to draw conclusions about patient experience and behavior with few qualitative studies (such as [21, 38, 40]) to provide a more nuanced understanding of the opportunities and barriers patients face accessing and using SHI.

### Policy context

#### The Ghana National Health Insurance Scheme (NHIS)

Ghana rolled out its National Health Insurance Scheme in 2003, making it one of the oldest SHI schemes in sub-Saharan Africa. Since then, membership has grown from 1.3 million people in 2005 to over 11 million active subscribers across a country of 28 million people. At the end of 2015, active card-bearing membership of the NHIS was 42%, with 80% of Ghana’s population identified as ever registered with the Scheme [41].

NHIS enrollment is technically mandatory for all Ghanaians, although in practice enrollment is voluntary for those working outside the formal sector [42]. Among formal sector employees who contribute to the the national pension scheme, a mandatory 2.5% deduction is made on their social security contributions to cover premium payments. Adults (18–69 years) in the informal sector pay an annual premium of between 15 Ghana Cedis (about four U.S. dollars) and 50 Ghana Cedis (about 10 U.S. dollars) depending on the district of registration. Children under 18 years, pregnant women, the elderly (≥70 years), pensioners, and indigents are exempted from paying the premium; this amounts to exemptions for more than two-thirds of NHIS members [43].

The NHIS is meant to cover all Ghanaians with the same package of inpatient and outpatient services. According to the NHIS website (www.nhis.gov.gh), this package includes outpatient services, such as consultations for malaria, respiratory infections, diarrhea, and anemia, along with ultrasound and x-ray testing, and a select list of prescription drugs. It also includes inpatient services, such as surgical operations and select cancer treatments, emergency and maternity services, eye care, and oral health. The package does not cover antiretroviral treatment for HIV, prosthetics, or cosmetic surgery, among others. Evidence suggests that this package has been successful at protecting households against catastrophic health expenditures (CHE) [44].

Ghana’s health system encompasses an estimated 3500 public, private, and faith-based health care facilities. Fifty-seven percent of these facilities are public, 33% are private, and 7 % are operated by the Christian Health Association of Ghana (CHAG). However, the share of private facilities ranges regionally from 5.4% in the Northern region to 74.9% in the Greater Accra region. Health facilities include Community-based Health Planning and Services (CHPS) compounds, health centers, clinics, maternity homes, and hospitals. While all CHPS compounds and most health centers and district hospitals are public, most clinics, maternity homes, and certain categories of hospitals are private [43].

Under the NHIS, providers are reimbursed for services according to diagnosis-related groups, a measure introduced to contain costs, while drugs are reimbused on a fee-for-service basis [43]. These reimbursement rates are determined by provider type and the level at which a provider is classified (e.g. hospital or clinic; public or private). Under this system, larger facilities receive higher reimbursement rates based on the assumption that they have higher operating costs. Private facilities also receive higher reimbursement rates than their public counterparts, because public facilities receive government funds to cover basic expenses (e.g. staff salaries, rent) while private providers must cover all expenses themselves. Whether public or private, smaller facilities qualify for reimbursement for fewer services and drugs; a system that is meant to discourage less qualified providers from providing services for which they have not been trained and are not equipped. However, widespread delays in reimbursement have created financial difficulties for providers [42]. Such difficulties may be magnified for providers in private practice who cannot depend on additional government funding to cover basic expenses. Some evidence suggests that providers are then shifting costs related to delayed or inadequate reimbursement to clients by charging them out of pocket for services that should be covered by the NHIS [40]. While clients complain about these charges and, as noted above, sometimes report discontinuing their NHIS enrollment because it covers so little (in practice), it is unclear if they seek out other providers, particularly in the public sector, as a result.

#### The Kenya National Hospital Insurance Fund (NHIF)

While the Kenyan NHIF was founded in the 1960s, its scope was quite limited, as it covered only civil servants for both inpatient and outpatient services, and offered only inpatient coverage to those working in formal sector jobs outside of the civil service. Recently, the NHIF has begun to expand rapidly, offering both inpatient and outpatient coverage to all formal and informal sector workers beginning in 2015 [13, 45]. The outpatient package includes general consultations, lab tests, x-rays, and minor surgical services, among others. Inpatient services covered include consultations, accommodation, radiology services, and operating theater charges. The NHIF also offers specialty packages to cover kidney care, oncology services, and substance abuse rehabilitation services, as well as a free package for maternity care. According to the NHIF’s 2018 annual report, the scheme now covers over 24 million Kenyans, with growth from 4.7 million principal beneficiaries in 2013 to 7.6 million in 2018 [46]. However, health insurance coverage in Kenya is generally low with only 19% of the population covered. While the NHIF is the main insurer in Kenya, it covered only 16% of the total population as of 2016 [47].

Under the NHIF system, enrollment is compulsory for civil servants and other formal sector workers, while those in the informal sector and retirees must enroll themselves. Similar to the Ghanaian NHIS, those working in the formal sector pay their premiums through monthly automatic deductions that are charged according to a sliding scale based on income level. Employers are required to match their employees’ contributions. Informal sector workers pay a monthly premium of 500 KSH ($5 USD) out of pocket. The government also contributes to care through various social programs, such as the free maternity scheme (Linda Mama) and insurance coverage for people living with severe disabilities, the elderly, and ophans and vulnerable children. A policy paper by Kenya’s Ministry of Health indicates that close to 6.2% of Kenyans spend over 40% of their non-food expenditure on health, which constitutes catastrophic health expenditure levels [48]. Indeed, despite increasing insurance enrollment and government-subsidized services, out of pocket health expenditure in Kenya remains high. Public facilities remain under resourced and patients often have to seek out specialized care (e.g. diagnostics, pharmaceuticals) in the private sector at a cost. Evidence shows that private providers in Kenya may charge well more than double the prices charged in public facilities for basic services, such as diagnostic testing and medication for hypertension [49].

Kenya has a mixed health market and, while the bulk of Kenyans still access care at public facilities, the private sector is growing rapidly. As of 2018, there were 10,820 health facilties in Kenya; 49% of these were public and 40% were private facilities with the remainder of the health market covered by faith-based and nongovernmental organizations [36]. It is estimated that in 2013, 42 and 44% of the population were receiving healthcare services at private facilities for outpatient and inpatient services respectively [50].

Among those health facilities that are NHIF-accredited, whether public or private, inpatient services are reimbursed on a fee-for-service basis with reimbursement rates based on provider level and type [51]. However, unlike the Ghanaian system, outpatient services are prepaid on a capitation system. Under capitation, clients enrolled in NHIF must register with a specific facility where they will receive all of their primary care. The NHIF puts out calls twice a year (May/June and December) for those who may want to change their registered facility. This requires submitting a change of medical facility form and waiting 1–3 months for the change to take effect with an additional waiting period before services can be accessed. However, enrollees can also make this change at any time themselves by accessing the form online [51].

Under capitation, accredited providers receive a lump sum payment from the NHIF on a regular basis that is based on the number of clients registered to their facility, regardless of whether or not each client seeks service. This system is meant to create a financial risk pool within each facility such that costs average out across those clients who use health services very little or not at all, and those who require more extensive services [52]. It also is meant to increase healthcare quality by promoting competition between accredited facilities vying for registered clients [53]. In theory, this system could incentivize clients to register with larger facilities that offer more comprehensive services, thereby consolidating the healthcare market around these larger providers, but some evidence suggests this may not be the case [54].

## Methods

This paper draws from two separate datasets collected under the African Health Markets for Equity (AHME) program. AHME, which was initiated in 2012 and concluded in 2019, was an initiative that aimed to increase access to quality primary care through private providers for low-income clients in Kenya and Ghana. The AHME partnership included Marie Stopes International, Population Services International, and the PharmAccess Foundation. Past partners included the International Finance Corporation, Society for Family Health, Nigeria, and the Grameen Foundation. AHME worked through social franchises, networks of providers that apply the principles of commercial franchising to health services [55], to provide a package of quality improvement and financing interventions. The participating franchise networks included the Amua and BlueStar franchises operated by Marie Stopes Kenya (MSK) and Marie Stopes Ghana (MSIG) respectively, and the Tunza franchise operated by Population Services Kenya (PS Kenya). The AHME intervention package included: social franchising; SafeCare, a step-wise quality improvement program managed by the PharmAccess Foundation; the Medical Credit Fund, a business training and loans program also managed by the PharmAccess Foundation; and SHI accreditation assistance. AHME also provided support for activities to identify and enroll low-income populations into the SHIs.

While we draw on both qualitative and quantitative data in this paper, it is not a mixed methods paper as such. The datasets analyzed in this paper were derived from two separate studies conducted within the context of the AHME program. One study was a qualitative evaluation of AHME, while the other was an analysis of client perspectives meant to improve service delivery at AHME supported clinics. Following Yin [56], we believe that because these studies were designed separately with different purposes and different research questions, the analysis that results from putting these two datasets in conversation with each other is not truly mixed methods. Rather, this is a qualitative analysis paper that seeks to integrate some quantitative findings in what Greene, Caracelli and Graham [57] call a “complementary” way; in order to elaborate, enhance, or clarify the findings of one study with the findings of the other.

### Study setting & sampling

#### The AHME qualitative evaluation

The qualitative dataset analyzed below was collected as part of the University of California San Francisco’s (UCSF) Qualitative Evaluation (QE) of AHME in both Ghana and Kenya. This dataset consists of 106 semi-structured exit interviews conducted with clients at both AHME-supported facilities and matched non-AHME facilities.

The data below were collected in two rounds: 2013 and 2017 to align with the original AHME project timeline. While this timeline was later amended, data collected following the 2017 round of data collection had not yet been analyzed at the time this paper was written. During each round of data collection, the QE team obtained lists of AHME-supported clinics from the project partners; in Round Two (2017) of data collection, these lists also included providers who had been contacted to join AHME, but had declined. These “matched” providers were located in healthcare facilities similar in size and location to the AHME-supported clinics and also met the criteria for franchising, making them an opportune point of comparison against the AHME-supported clinics.

Drawing from these lists, the QE team used a purposeful criterion sampling strategy [58] to design a sample that included providers with a range of experiences with the AHME intervention package. In 2017, this included the matched non-AHME providers who had no experience with the interventions. Interviews were conducted across six regions in Kenya (Nairobi, Eastern, Coast, Central, Rift Valley, Kajiado) and three regions in Ghana (Greater Accra, Volta, Ashanti). Since AHME’s reach expanded over the course of the project, the study regions were largely chosen opportunistically depending on where program activities were focused. In some cases, a region was chosen specifically because SHI programs were expanding or being tested in that area, such as the NHIS’s capitation pilot in the Ashanti region.

The AHME QE was part of a larger mixed methods evaluation that included a Randomized Controlled Trial (RCT) in Kenya. The QE team therefore sought to align the sampling criteria for client exit interviews with those laid out by the RCT. These criteria included: gender (women only); age (between 18 and 49 years of age); and number of children (interviewees were required to have at least one child aged 5 years or less). Potential respondents also had to be exiting one of the selected AHME or matched clinics and the QE team aimed to sample both NHI-enrolled and non-enrolled patients equally in the 2017 round of data collection. However, NHI-enrolled patients were over-sampled in Ghana, because NHIS coverage is relatively high and most clients screened for participation were enrolled. In 2017, the QE team purposefully over-sampled patients exiting clinics located in low-income areas to better capture data related to AHME’s overarching goal of reaching poor populations with quality, affordable care (Table 1).

**Table 1:** AHME Qualitative Evaluation Patient Sample

		Round 1 (2013)		Round 2 (2017)	
		Kenya	Ghana	Kenya	Ghana
**Total**		**26**	**20**	30^*^	**30**
**Franchised clinics**		**26**	**20**	**18**	**23**
	Tunza (PSK)	19	N/A	9	N/A
	Amua (MSK)	7	N/A	9	N/A
		*NHI-enrolled*	*NHI-enrolled*	*NHI-enrolled*	*NHI-enrolled*
		13	N/A	14	27
^1^**Non-franchised clinics**		**N/A**	**N/A**	**8**	**7**

* While a total of 30 clients were interviewed in Round 2 in Kenya, identifying information for four of these clients was not transmitted back to the research team at UCSF and cannot be recovered. We believe we are missing identifying information for clients from two franchised and two non-franchised facilities

#### AHME client exit interviews

In addition to the qualitative evaluation data, this paper uses internal data from client exit interview surveys conducted at AHME facilities at two different points in 2018 for a total sample size of 4042 client exit interviews. These were cross-sectional surveys of social franchise clients at three networks supported by AHME. See Table 2 for an overview of the sampling pattern used to conduct the client exit surveys. The target sample size for each network, based on historic client flow data, was a minimum of 214 clients, across a randomized sample of 40 franchise sites per network for the first round of data collection, while one AHME partner (MSIG) conducted a census of all 107 of their facilities in the second round of data collection. The random sample was generated using a sample generator built in Microsoft Excel; this involved using Excel’s random number generation function and comparing this list to a numbered list of facilities. The overall sample size was set to account for a non-response rate of 10%. It was estimated to achieve a margin of error of +/− 10%, around point estimates of 50%, at a significance level of 95% with an estimated design effect of two.

**Table 2:** Sampling Patterns and Sample Sizes for AHME Client Exit Surveys

Partner	Round 1 (Feb. - May 2018)	Round 2 (Dec. 2018)
MSK	All clients for three days	All clients for two days
	724 interviews	414 interviews
PS Kenya	All clients for three days	N/A
	604 completed interviews	
MSIG	All but every fifth client for three days	All clients for one day at all facilities*
	1509 interviews	791 interviews

*A census of all 107 facilities, not a sample of 40 facilities

Data collectors were instructed to interview all clients receiving a franchised service at selected sample sites for 3 days at MSK and PS Kenya, and all but every fifth client receiving a franchised service for 3 days at MSIG. During the second round of data collection, all clients at MSK facilities were interviewed across 2 days. Skip patterns and number of interview days varied across partner organisations in line with average client flow per social franchise site to ensure that the minimum sample of 214 was reached. Franchised services include: Family Planning, treatment and testing for sexually transmitted infections, malaria testing and treatment, treatment of illness in a child under five, antenatal and postnatal care, and safe delivery services (PS Kenya only). Clients were asked questions on health insurance status and usage as part of a larger survey on service use, marketing, and client satisfaction.

### Data collection

#### The AHME qualitative evaluation

The QE team partnered with the research organization Innovations for Poverty Action (IPA) to collect data in both Kenya and in Ghana. IPA recruited local field interviewers in each country and these interviewers were jointly trained by the UCSF QE team and IPA. Data collection took approximately 1 month in each country during each round. Individual interviews lasted approximately 30 min each. To recruit participants, interviewers obtained permission from providers to approach their clients for screening eligibility screening as they prepared to leave the facility. If a client met the screening criteria and also agreed to an interview, they went through the informed consent process with the interviewer and immediately interviewed in an area of the clinic that was relatively private and quiet. In both rounds of data collection, clients were asked a series of questions related to their healthcare experiences, health-seeking behaviors, and knowledge of the local healthcare landscape. In Round Two (2017) of of data collection, clients also were asked about their experiences with and knowledge about SHI. An additional file details the questions that were asked of each participant in Round Two of interviews [see Additional file 1].

Interviews were recorded using digital recorders in the language the respondent was most comfortable using. Interviews were conducted by local interviewers who were fluent in both English and the appropriate local language, and interview guides were first developed in English and then professionally translated into the local language (Swahili in Kenya and Twi in Ghana) to ensure consistent and accurate translation. Following the completion of data collection in each round, recordings were simultaneously translated and transcribed simultaneously by a team of professional transcriptionists.

#### AHME client exit interviews

Data collection took place at MSIG in February 2018, achieving a final survey sample size of 1509 and again in December 2018 with a final sample size of 791 interviewees for a total of 2300 client exit interviews conducted at BlueStar facilities. MSK data collection took place in May 2018, with a final survey sample size of 724 and again in December 2018 with a final sample size of 414 interviewees for a total of 1138 client exit interviews across the two rounds at Amua facilities. These data were collected from 39 clinics in May and from the target 40 facilities in December; MSK was not able to reach one provider to conduct exit interviews at their clinic in May, which decreased the target sample for this round. PS Kenya data collection took place in April 2018, with a final survey sample size of 805. While PS Kenya has plans to conduct a second round of surveys, this data was not available in time to be included in this paper. Data presented below is based on responses from 604 of the PS Kenya interviews, as not all respondents from PS Kenya answered all questions.

### Data analysis

#### The AHME qualitative evaluation

IPA staff fluent in both English and the local language back-checked interview transcripts before transferring them to the UCSF QE team via an encrypted server. The QE team then coded these transcripts with some assistance from IPA using a widely-used qualitative analysis software package (Atlas.ti). Taking an inductive, thematic approach to coding and analysis, the QE team developed an initial coding scheme based on thematic coding of a sub-set of interviews from each country, and reviewed this scheme both internally and with IPA to ensure consistency in application of codes. In order to allow for emerging research priorities, codes were refined across rounds of data collection. Additional information on the UCSF QE team’s methodology can be found in a previous publication [59].

#### AHME client exit interviews

An SPSS cleaning syntax developed specifically for this survey was used to clean the data and cases with missing values were omitted from the analysis. Data analysis was conducted using Stata 14. The analysis presented below includes only descriptive statistics. This is because a descriptive analysis allows for characterization of the distribution of a characteristic across a given population, whereas other statistical analyses, such as multivariate analysis, are suitable for causal inference and the determinants of an outcome. Since the quantitative data included in this paper is meant to reflect respondents’ experiences with SHI, rather than to make claims about the reasons behind their behavior with regards to SHI, we believe descriptive analysis is the most appropriate method to use in this case.

### Ethical review

The QE team received initial approval for the AHME evaluation with “Exempt” status from UCSF’s Institutional Review on 13 June 2013. Ethical approval also was obtained from the Ghana Health Services Ethical Review Committee (ERC) and the Kenya Medical Research Institute (KEMRI). Prior to each round of data collection, the QE team received approval from all three ethical review boards for any changes made to the research protocol. Approvals for Round 2 (2017) of data collection were received: on 12 December 2017 from UCSF, 16 November 2017 from the ERC, and 17 January 2017 from KEMRI.

The ethical approval process was not required for AHME’s internal Client Exit Interviews.

## Results

### Choosing when and where to seek care

In most cases, clients interviewed for the Qualitative Evaluation (QE) indicated that they sought care more frequently with SHI coverage because it was more affordable.

*Because with the card, if I go anywhere and I’m registered – when I’m sick, I just take it along with me.****With that you don’t fail to go to the hospital because of lack of money, right?*** (Patient at a Bluestar clinic, Ashanti, Ghana).

In some cases, patients also felt that having SHI coverage gave them access to a wider variety of providers, most notably private providers, than they would have if paying out of pocket. This was particularly appealing given that clients often stated a preference for private providers for the caring and respectful treatment they received at private facilities, as well as private providers’ efficiency compared to public providers. During the final round of QE interviews (2017), some clients in Kenya were especially grateful for the access their SHI coverage afforded them while the country’s public health doctors went on strike.

*You know, right now, all the doctors are on strike and so even if there is…the medical services in the government hospitals are not good****. Even when we went there, we were forced to come to this private hospital. Because we went there and the medical services were not good****.* (Patient at an Amua clinic, Eastern, Kenya).

Although we have not yet analyzed data collected for the QE in 2018, a nurses’ strike may have similar effects by putting more strain on a public health system that is already under-resourced.

However, while clients reported that they were more likely to seek care when covered by SHI and appreciated the access it gave them to a wider variety of providers, having the coverage did not necessarily affect their provider choice. In Ghana, where the NHIS has been widely available to the population for a longer period of time than Kenya’s NHIF, the AHME client exit survey found that most BlueStar clients were enrolled in NHIS. However, when asked their main reasons for selecting the facility where they were interviewed, clients most commonly responded that the facility was “nearby” (25% in February; 22% in December), “recommended by a friend” (16% in February; 25% in December), had a “good reputation” (15% in February; 28% in December), or was in a “convenient location” (15% in February, 5% in December). Thus, despite the high prevalence of franchise clients with NHIS coverage in Ghana, provider choice did not appear to be driven by NHIS accreditation status.

Similarly, accreditation status was not a strong predictor of provider choice in Kenya. Unlike in Ghana, clients participating in the AHME exit survey in Kenya were asked why they did not choose to see an alternative provider and one of the options given was “[Provider] doesn’t accept NHIF.” None of the clients exiting MSK facilities chose this response. Three percent of PS Kenya clients (4 cases) answered “doesn’t accept health insurance” when asked a similar question. The most common reasons why clients chose the facility where they were interviewed were: “good reputation of facility;” “offers quality services;” “nearby;” “convenient location;” and “good reputation of provider.”^1^ “Accepts insurance” was not one of the options on the survey administered to MSK clients, but only 3% (16 cases) of PSK clients answered “accepts health insurance” when asked why they chose the facility they attended that day.

Unlike in Ghana, where a relatively large percentage of the population is enrolled in NHIS and most providers are accredited, NHIF coverage in Kenya has only started to expand more recently. As a result, enrollment is still low compared to Ghana and fewer providers are NHIF-accredited. Among clients who participated in the exit survey in Kenya, only 30% of MSK clients were enrolled in NHIF while this number was higher (54%) among PS Kenya clients.^2^ Although there was a statistically significant increase in the percentage of MSK clients who reported being enrolled between the May and December rounds of data collection, the majority (57%) of those surveyed were still not enrolled. Due to the lower numbers of accredited providers in Kenya, provider choice is more limited for those who wish to use their NHIF coverage; this likely affects the extent to which patients consider a provider’s accreditation status when choosing where to seek care. Indeed, in the QE interviews some clients in Kenya reported that they had NHIF coverage and paid for it monthly, but rarely used the coverage because they did not seek out an accredited clinic.

*Interviewer: And out of the five visits you have gone to the hospital, how many times have you used NHIF?*

*Respondent: I have only used it once.*

*Interviewer: Why?*

*Respondent: Because I don’t frequently go there.*

*Interviewer: Where is that?*

*Respondent: Where they use the NHIF. You know I don’t know if here [at the Tunza clinic] they use the NHIF. And I only know of there.* (Patient at a Tunza clinic, Central, Kenya).

Similarly, of those MSK clients participating in the exit survey who were covered by NHIF, only a minority (21%) tried to use it to pay for services received that day.^3^ Among PSK clients, 14% of clients answered “no NHIF facilities in my location” when asked why they hadn’t enrolled. However, the most common response to this question was “Lack of adequate information to make a decision” (41%), which aligns with results from the qualitative data suggesting that Kenyans were generally less informed regarding what health insurance is and how to use it than clients interviewed in Ghana. In addition, the QE data suggests that Kenya’s capitation system may discourage patients from seeking care at accredited facilities if they are not registered at a clinic that is convenient for them.

### Trusting the system

While many Kenyan clients did not have NHIF coverage, among those interviewed for the QE in both countries patients generally felt secure using their coverage and trusted that they would receive quality services when using their SHI card to pay.

*Interviewer: Oohh…okay, is it that when you go to the hospital with the NHIF card the doctors look at you or treat you differently from those who don’t have the NHIF card?*

*Respondent: No. We…we are all equal.*

*Interviewer: Whether you have the card or not?*

*Respondent: Yeah.* (Patient at a Tunza clinic, Rift Valley, Kenya).

Client exit surveys conducted in Kenya support this sentiment, with the majority of enrolled clients who tried to use their insurance at both MSK and PS Kenya facilities reporting that NHIF covered all of their services on the day they were surveyed.^4^ Similarly, few clients participating in QE interviews reported that they had trouble using the NHIF card to pay for services or had been refused services when trying to pay with NHIF in the past.

However, exit surveys in Ghana, where QE interviews also suggested that clients were more hesitant to trust the system than their counterparts in Kenya, revealed that either clients misunderstand the service package, providers are misunderstanding and misrepresenting the package, or some combination of the two. When clients exiting BlueStar clinics were asked why they didn’t try to use their insurance coverage to pay for services that day, why it wasn’t accepted, or why it was only accepted for some services, across both rounds of data collection the vast majority of respondents said it was because the service they received was not covered. Most of these clients received antenatal care, malaria services, or treatment for illness in a child under five years, all of which are covered under the NHIS.^5^

### Challenges with access

Despite generally positive attitudes toward both the NHIS and NHIF, clients in both countries were sometimes deterred from using their coverage because providers still levied out-of-pocket charges for specialty treatments and drugs, or refused to accept the insurance at all. This problem was especially prevalent in Ghana, where financial issues at the NHIS have led to provider reports of reimbursement delays up to one year [59]. Indeed, exit surveys conducted at BlueStar facilities revealed that private providers in Ghana may not be accepting NHIS as payment even when they are empanelled. During both rounds of client exit surveys, the majority of clients who said they could not use their NHIS coverage because the facility they were attending did not accept NHIS were actually accessing services at an empanelled facility. While lack of knowledge regarding facility accreditation status was clearly a barrier to patients using their NHIS coverage in this instance, our QE data also suggests some clients were aware that their access to healthcare was still limited under NHIS. This both undermined patients’ trust in the system and discouraged them from enrolling with or renewing their NHIS enrollment altogether.

*Respondent: Mm, me, to me, right now I don’t have health insurance. I have the card all right, but it’s at home. When I visit the hospital, I go with money.*

*Interviewer*: *Okay, okay, okay, why?*

*Respondent*: *Because when I even possess the health insurance it covers nothing. Mm.* (Patient at a Bluestar clinic, Ashanti, Ghana).

As noted above, most patients enrolled with NHIF in Kenya were able to use their coverage at accredited facilities. However, during the May 2018 round of exit surveys, the most common reason MSK clients gave to explain why they had not tried to use their NHIF coverage was that they were visiting a provider who did not accept NHIF (34%); 11% of the clients who gave this answer accessed services at empanelled facilities.^6^ The second most common reason MSK clients gave when asked why they hadn’t used NHIF coverage to pay for their services on the day they were surveyed was that the service was not covered (24%). However, 27% of the clients who gave this answer were receiving only antenatal care, prenatal care, malaria treatment, and/or treatment for an illness in a child under 5 years, all of which are covered under NHIF.

Notably, the percentage of MSK clients claiming they hadn’t paid with NHIF because the facility did not accept it dropped sharply during the December 2018 round of exit surveys to 11%; a statistically significant decrease. During this round, the most common responses to this question were: “other” (20%); “I am not registered at this facility [under capitation]” (19%); and “service received not covered by insurance” (16%), although the majority of this 16% did in fact receive a covered service. This encouraging and significant change likely reflects both the rapidly shifting healthcare landscape in Kenya as well as internal changes to MSK’s Amua franchise network. Over the past several years the NHIF has been expanding both the coverage it offers (e.g. the Supa Cover scheme for informal sector workers introduced in 2015 and the Edu Afya scheme to cover secondary school students introduced in 2018) and the number of facilities accredited to provide covered services. At the same time, both MSK and PS Kenya have been making concerted efforts under the AHME program to assist network providers with the accreditation process, and to increase the number of Amua and Tunza facilities that accept NHIF. This assistance is accompanied by demand generation activities to inform community members that they can use their NHIF coverage at Amua and Tunza facilities.

## Discussion

Our findings suggest that SHI has potential to increase access to primary healthcare, including a wider variety of providers and services for clients in both Ghana and Kenya. Indeed, many of the clients interviewed appreciated that insurance coverage made healthcare more affordable and reported seeking care more frequently when covered with SHI. Clients also noted that the coverage gave them access to a variety of providers, especially those in the private sector, who they tended to prefer over public providers. Further, clients reported few difficulties using their SHI card to receive care and often felt that they were given the same treatment as those paying out of pocket. This suggests that clients generally trusted in the SHI system over all, although there were notable exceptions in Ghana, where the NHIS has recently been under-funded. As previous studies have found that SHI systems sometimes have trouble achieving UHC because patients lack trust in the public health system [12], this study therefore suggests that accrediting private providers into the SHI scheme may be one way in which to address this challenge. However, governments should be sure to adequately finance these endeavors and also provide reimbursements for private provider that align with their actual costs [60, 61].

However, our data reveals several challenges SHI schemes in both countries will need to overcome in order to advance toward UHC. In both countries, clients and providers would benefit from education on how insurance coverage works, how to use it, and what is included in the SHI package. As noted in previous studies [19, 38], we found that clients sometimes were charged for services that should have been covered by their insurance. While clients rarely appeared to know they had been charged inappropriately and their healthcare-seeking behavior did not seem to be affected as a result, any charges are nevertheless a barrier to care for patients living in poverty, whether or not these charges are appropriate. Indeed, lower income patients may struggle to cover such basic expenses as the cost of transportation to the clinic, making any additional charges particularly cumbersome [62, 63]. It is therefore important that patients know their rights in relation to their insurance coverage, which other studies have shown is challenging to accomplish, even with public information campaigns [64]. However, one study conducted in Burkina Faso has shown some success educating community members about a community-based health insurance scheme using a combination of multiple media channels to disseminate information with participation from community leaders [65].

Further, because so many clients lacked information about their rights and benefits under SHI, it is all the more critical that providers are educated and held accountable for misusing the system. We have suggested elsewhere that monitoring mechanisms built into SHI schemes might be one way to accomplish this [59]. However, just as a lack of sustainable funding has been cited as a major challenge for SHI systems to overcome in general [15], current under-funding of the Ghana NHIS puts private providers in a position where they cannot cover basic expenses with NHIS alone. These providers therefore feel they have no choice but to charge clients in order to maintain a viable business [40]. In this case, the National Health Insurance Authority in Ghana must take the additional step of ensuring the system’s financial stability and sustainability in order to minimize providers’ incentive to charge patients for services that should be covered under the SHI.

Finally, although clients appeared to value their SHI coverage, it was surprising that they did not prioritize a provider’s SHI accreditation status when choosing where to seek healthcare. In order for SHI to be effective, clients must first prioritize using the coverage. This problem may resolve itself to some extent in Kenya as more providers become empaneled and Kenyans find themselves with greater access to facilities that will accept their NHIF card. However, given the low prevalence of providers outside major urban areas in both Ghana and Kenya [66, 67] much of the population, and particularly those living in poverty in rural areas, has little access to *any* formal healthcare. In this context, SHI accreditation status becomes an irrelevant decision-making point and it is no surprise that clients tended to prioritize facility location when choosing where to seek health services. Since private facilities provide a significant proportion of all health services in both countries [45, 68, 69], the NHIF and NHIS should therefore encourage private providers to become accredited in order to expand general access to facilities that accept SHI coverage. In addition to efforts already in place to expand access to primary care in rural areas, such as the Ghana Health Service’s Community-Based Health Planning and Services (CHPS) compounds, governments should also update incentives to both public and private providers to work in rural and remote areas [70, 71]. This would not only expand healthcare access generally in these areas, but if the SHI systems also focus on accrediting these providers, such facilities would be both geographically and financially accessible to a larger percentage of the population, making UHC a much more achievable goal.

## Conclusions

Ensuring enrollee buy-in is critical to the success of SHI schemes that aim to move countries like Ghana and Kenya toward UHC. Our findings suggest that enrollees in both countries valued their SHI coverage. However, SHI coverage had little effect on provider choice, particularly in Kenya. This suggests that patients continue to prioritize other factors, such as geographic accessibility, over a provider’s SHI accreditation status. To address this issue on the healthcare supply side, we recommend increasing the number of private providers accredited into SHI schemes in both countries and updating incentives for both public and private providers to work in underserved rural areas. In addition, governments should adequately align funding for SHI schemes with the actual costs incurred and needs of providers, paying particular attention to the differentiated funding needs of providers in the public and private sectors. Finally, SHI enrollees require further education about the benefits they can expect to receive under SHI. In combination with increased monitoring from the government side, this should help to hold providers accountable for charging patients incorrectly and, in turn, bring healthcare costs down on the demand side. Taken together, these recommendations can help to ensure both a more comprehensive reach by SHI schemes, and greater enrollee allegiance to and trust in these schemes, bringing countries like Kenya and Ghana closer to achieving UHC through SHI.

## Supplementary information

**Additional file 1.**

## Data Availability

The datasets supporting the conclusions of this article are not publicly available. The informed consent process for the AHME qualitative evaluation did not include a provision for sharing data and participants were explicitly promised that their data would be destroyed upon completion of the study. However, the data collection instruments (interview guides) have been appended to this article and the analysis tools (codebook for Atlas.ti qualitative data analysis software) can be made available by contacting the corresponding author (Lauren.Suchman@ucsf.edu). Relevant excerpts from the transcripts are included in the body of the paper. Marie Stopes does not make its study instruments publicly available. However, more information about the study instrument can be obtained by contacting Catherine Verde Hashim at Catherine.Verdehashim@mariestopes.org.

## Abbreviations

AHME: African health markets for equity
CHAG: Christian health association of Ghana
CHE: Catastrophic health expenditures
CHPS: Community-based health planning and services (Ghana)
ERC: Ghana health service ethics review committee
IPA: Innovations for poverty action
KEMRI: Kenya medical research institute
LMIC: Low- and middle-income countries
MSIG: Marie Stopes International Ghana
MSK: Marie Stopes Kenya
NHIF: National hospital insurance scheme (Kenya)
NHIS: National health insurance scheme (Ghana)
PS Kenya: Population services Kenya
QE: Qualitative evaluation
RCT: Randomized controlled trial
SHI: Social health insurance
UCSF: University of California San Francisco
UHC: Universal health coverage

## Footnotes

1 This question was only included in the May 2018 round of data collection, but not in the shorter version of the survey that was administered in December.

2 We found a correlation between NHIF coverage and wealth in both the PS Kenya and MSK datasets. Since PS Kenya clients tend to be wealthier, it is not surprising they are also more likely to be enrolled in NHIF.

3 In contrast to Ghana, where most (77%) clients with NHIS coverage tried to use it to pay for their visit.

4 However, note that only a minority of clients who reported that they were enrolled in NHIF actually tried to use their coverage at the clinic. In May, this was 21% of clients who reported being enrolled and 28% in December. At PSK clinics, usage was slightly higher at 37%.

5 Some clients in this group received more than one service, so it is not accurate to add up these percentages. However, we can confirm none of the clients who received these three services also received a family planning or safe abortion service. Safe abortion is not covered by the NHIS while family planning is technically covered, but in practice providers are not reimbursed for family planning services.

6 While all clients included in the MSK exit interviews were asked why they hadn’t used NHIF coverage to pay for their care that day, among PSK clients, only those whose NHIF coverage hadn’t been accepted were asked why this had happened. Since only 19 people fell into this category, we cannot draw similar conclusions about NHIF usage among clients at PSK facilities.
