# Correction to: Seeking care in the context of social health insurance in Kenya and Ghana

**DOI:** 10.1186/s12889-020-09076-8

**Published:** 2020-06-15

**Authors:** Lauren Suchman, Catherine Verde Hashim, Joseph Adu, Rita Mwachandi

**Affiliations:** 1 Institute for Global Health Sciences, University of California San Francisco, San Francisco, CA USA; 2 Marie Stopes International, London, UK; 3 Marie Stopes Ghana, Accra, Ghana; 4 Population Services Kenya, Nairobi, Kenya

**Correction to: BMC Public Health (2020) 20:614**

**https://doi.org/10.1186/s12889-020-08742-1**

It was highlighted that the original article [1] contained an incorrect statement in the Ethics approval and consent to participate section. This Correction article shows the correct Ethics approval and consent to participate section.

**Ethics approval and consent to participate**

The QE team received initial approval for the AHME evaluation with “Exempt” status from UCSF’s Institutional Review on 13 June 2013. Ethical approval also was obtained from the Ghana Health Services Ethical Review Committee (ERC) and the Kenya Medical Research Institute (KEMRI). Prior to each round of data collection, the QE team received approval from all three ethical review boards for any changes made to the research protocol. Approvals for Round 2 (2017) of data collection were received: on 12 December 2017 from UCSF, 16 November 2017 from the ERC, and 17 January 2017 from KEMRI.

Ethical approval for Round 1 of AHME’s Client Exit Interviews was obtained from Marie Stopes International’s Ethics Review Committee (MSI ERC) on 25 August 2017, the Ghana Health Service Ethics Review Committee (GHS ERC) on 20 November 2017, and Amref Health Africa Ethics and Scientific Review Committee (Amref ESRC) on 30 April 2018. Approvals for Round 2 were received from MSI ERC on 10 September 2018 and GHS ERC on 12 November 2018. The approval received from Amref ESRC on 30 April 2018 covered both Round 1 and Round 2 of data collection in Kenya.
