# Supplementary material for: Seeking care in the context of social health insurance in Kenya and Ghana
Source: BMC Public Health. 2020 May 4;20:614. doi: 10.1186/s12889-020-08742-1 (PMC7197151; doi:10.1186/s12889-020-08742-1)
Supplement: Supplementary file 1 — Additional file 1. [file 12889_2020_8742_MOESM1_ESM.pdf]

In-Depth Interview Field Guide: Franchise Patient - Round 2

Study Title: Qualitative Process Evaluation of the AHME Partnership
IRB No: 13-11045
Version: November 2016

Session ID: [__ __] [__ __ __]

Date (DD/MM/YY): ___ /___ / ___

Name of Interviewer: ____

Name of Clinic: ____

Before the Interview

---

1. ***Introduce yourself and the study [READ INTRODUCTION SCRIPT]***

2. ***Make sure the participant is eligible***
   a. Is the participant female? ☐ YES ☐ NO
   b. Is she between 18-49 years of age? ☐ YES ☐ NO
   c. Does she have at least one child who is between 0-5 years of age? ☐ YES ☐ NO
   d. Did she visit the clinic today for her child? ☐ YES ☐ NO
   e. Did she visit the clinic today for herself for a reproductive or maternity reason? ☐ YES ☐ NO
   f. Is she covered by NHIF? ☐ YES ☐ NO

3. ***Obtain informed consent [READ CONSENT SCRIPT]***
   a. Did you answer any questions she had? ☐ YES ☐ NO
   b. Did she agree to participate? ☐ YES
      ☐ NO → *STOP INTERVIEW*
   c. Did you give her a copy of consent form? ☐ YES ☐ NO

4. ***Obtain permission to tape record***
   a. Did she give permission to be tape-recorded?
      ☐ YES → *TURN ON RECORDER*
      ☐ NO → *THANK HER AND ASK TO LEAVE*

5. ***Turn on tape recorder and say***
   a. Session ID
   b. Interviewers present

Interview

First I'd like to know a bit about your visit to the clinic today.

1) Did you visit the clinic today for yourself or for your child?
    a. Compared to other clinics, what made this one seem like a good place to go?
        i. **<u>If patient was referred</u>**: Who referred you to this clinic and why? *<u>Probe</u>*: If you were told this was a "good clinic", why do you think they said that?
    b. What health reasons normally bring you to this clinic?

Let's talk about health services for your child.

2) What are the illnesses your children most commonly suffer?

3) What is the first thing you usually do at home when you notice your child is sick?

4) How do you know when you need to seek treatment for your child outside the home?

5) Where do you usually bring your youngest child for treatment when he or she is sick?
    a. Why is that a good place to seek treatment?

Now, thinking about your visit to the clinic today:

6) What do you think of your experience today at the clinic?

7) Is this clinic different from government health centers and hospitals?
    a. If so, how?
    b. Probe: Accessibility, drug supply, service available, staff, cost

8) Is this clinic different from other private clinics?

   a. If so, how?

   Probe: Accessibility, drug supply, service available, staff, cost

9) Is this clinic different from chemists or drug stores?

   a. If so, how?

   Probe: Accessibility, drug supply, service available, staff, cost

10) Is this clinic different from herbalists?

   a. If so, how?

   Probe: Accessibility, drug supply, service available, staff, cost

11) Are there any health services not offered in your community that you wish you had? What are they?

12) Have you heard of Tunza / Amua?

   a. **IF YES:** How did you hear about Tunza/Amua?
   b. **IF YES:** What do you know about Tunza / Amua?

I'd like to ask a few questions about NHIF.

13) What do you know about NHIF?

   Probe: who is eligible to have NHIF membership?

   a. When and where did you first hear about NHIF?
   b. What do you know about additional/special NHIF programs (e.g. Supa Cover)?
   c. Do you know about the process for applying to NHIF?
      i. **IF YES:** Could you describe the process to me?
      ii. **IF YES:** What is challenging about applying for NHIF?

14) Are you and/or your children registered with NHIF?

<u>IF YES</u> <u>continue to question 15:</u>

<u>IF NO</u>: Have you thought about registering?

<u>IF YES</u>: Why did you decide not to apply?

Probe: cost, time, registration process, doesn't know where to go

GO TO QUESTION 16

15) What has your experience with NHIF been like?

a. Is it easy to use?
    i. Out of every 5 visits you make to a hospital or clinic, how many times do you pay with NHIF?
    ii. Why?
b. What are the benefits of having NHIF?
c. Has a clinic ever rejected NHIF as payment for your care?
    i. **<u>IF YES:</u>** What was the reason?
d. Do you still have to pay for services when using your NHIF card?
    i. **<u>IF YES</u>:** Do you have additional insurance that you use for this?
e. Do providers treat you differently if you pay with NHIF?
f. Have you experienced any other challenges with NHIF?

16) Do you know of any services not covered by NHIF?

<u>IF NO</u>: skip to question 17

<u>IF YES</u>:

a. What are they?
b. How do you pay for those services?
c. How difficult is it for you to pay those costs?
d. Do you get those (services/medicines) from the same clinic you go to for care, or somewhere else? Why?

17) Do many people you know get health coverage from NHIF?

Probe: About how many out of 10 have NHIF?

a. What has it been like for your family members/friends/others you know to use NHIF?

18) How much did you pay for your visit today, including cost of drugs and any procedures?

a. What do you think of this cost?
b. Is this more, less, or the same as other health facilities?
c. **IF HAS NHIF:** Did NHIF pay for any of your healthcare costs today?
    i. **IF YES:** Did your ability to pay with NHIF affect your decision to come to this clinic?
    ii. **IF NO:** Why did you choose not to pay with NHIF today?
d. Are there other ways that you've paid for health services today or in the past?

Probe: vouchers, personal health insurance, in kind payments (maize)

Lastly I would like to tell you a story and hear your opinion.

19) Sometimes women face difficult decisions about pregnancy and maternity care. I would like to tell you a story about one such woman named Catherine. Catherine is 17 years old. She moved to Nairobi from her village a year ago for work. She starts dating her co-worker John, who is 19. After 4 months, Catherine learns that she's pregnant. Pretend that you are Catherine's friend and she has come to you for advice.

i. What do you advise her to do?
ii. Whom do you advise her to talk to? How should she talk to John about what to do?
iii. Where is she likely to go for care? Why?
iv. What is the best place for her to go for care?

Catherine is not in a position to care for a child right now. She decides to terminate the pregnancy.

i. What would you think of this decision? Why?
ii. Is there anywhere in this community she could get an abortion? If no, how far outside this community would Catherine have to go for this kind of care?

20) Is there anything else we haven't discussed about your healthcare that you think would be helpful for me to know?
Probe: your experience visiting this clinic; how you pay for healthcare; your experience with NHIF; healthcare in your community?

Thank you for participating in the study. We appreciate your time. We are nearly finished. I would just like to ask a few questions about your background.

TURN OFF RECORDER

Demographic Sheet: Franchise Patients

Session ID: [__]--[__][__ __] **Date (DD/MM/YY)**: ___ /___ / ___

No.	Question	Coding Categories
1	How old are you (in years)?	|__|__|
2	What is your marital status?	☐ 0. Single
		☐ 1. Married
		☐ 96. Other → please specify ______________________________
3	How many children do you have?	|__|__|
4	How many of your children are 5 years or less?	|__|__|
5	What is the highest level of education you have completed?	☐ 0. Never went to school or less than completing primary
		☐ 1. Primary education
		☐ 2. Secondary education or high school
		☐ 3. College or tertiary education (vocational, trade school, diploma, certificates)
		☐ 4. University (degree)
		☐ 5. Masters/doctorate
6	What is your monthly income	☐ 1. 10,000 (or below)
		☐ 2. 10,001 – 20,000
		☐ 3. 20,001 – 30,000
		☐ 4. 30,001 – 40,000
		☐ 5. 40,001 – 50,000
		☐ 6. More than 50,000
7	What is your ethnicity?	☐ 0. Kalenjin
		☐ 1. Kamba
		☐ 2. Kikuyu
		☐ 3. Kisii
		☐ 4. Luo
		☐ 5. Luhya
		☐ 6. Nubi
		☐ 7. Meru
		☐ 8. Borana
		☐ 9. Mijikenda
		☐ 10. Embu
		☐ 11. Maasai
		☐ 12. Somali
		☐ 13. Gabra
		☐ 14. Rendille
		☐ 15. Pokomo
		☐ 16. Ormo

		☐ 17. Giriama
		☐ 18. Taita Taveta
		☐ 19. Turkana
		☐ 20. Samburu
		☐ 21. Duruma
		☐ 22. Digo
		☐ 96 Other → please specify ______________________________
8	What is the main language you speak at home?	☐ 0. English
		☐ 1. Kiswahili
		☐ 2. Kalenjin
		☐ 3. Kamba
		☐ 4. Kikuyu
		☐ 5. Kisii
		☐ 6. Luo
		☐ 7. Luhya
		☐ 8. Nubi
		☐ 9. Meru
		☐ 10. Borana
		☐ 11. Mijikenda
		☐ 12. Embu
		☐ 13. Massai
		☐ 14. Somali
		☐ 15. Gabra
		☐ 16. Rendille
		☐ 17. Pokomo
		☐ 18. Orma
		☐ 19. Giriama
		☐ 20. Taita Taveta
		☐ 21. Turkana
		☐ 22. Samburu
		☐ 23. Duruma
		☐ 24. Digo
		☐ 96 Other → please specify ______________________________
9	What is your religion?	☐ 0. Catholic
		☐ 1. Protestant
		☐ 2. Adventist
		☐ 3. Muslim
		☐ 4. Buddhist
		☐ 5. Hindu
		☐ 6. Sikh
		☐ 7. Traditional
		☐ 8. Shintoism
		☐ 9. None

		☐ 96. Other → please specify ____________________
10	Where do you currently live?	Specify: ____________________<br><br>*[Leave codes blank – to be completed after]*
		☐ 0. Rural
		☐ 1. Peri-urban
		☐ 2. Urban
11	How long have you lived in your current location (in years)?	|__|__|
12	Before you lived in your current location, where did you live?	Specify: ____________________<br>*[Leave codes blank – to be completed after]*
		☐ 0. Rural
		☐ 1. Peri-urban
		☐ 2. Urban
		☐ 88. Not applicable – has always lived in same location
13	What was the reason that you moved to your current location?	☐ 0. Job
		☐ 1. School
		☐ 2. Family
		☐ 96. Other → please specify ______________
		☐ 88. Not applicable – has always lived in same location
14	At the clinic today, how long did you have to wait to be seen (in minutes)?	|__|__|__|
15	How much did you spend on today's visit (include cost of drugs, services, procedures, and consultation)?	Local currency:
16	How did you get to clinic today?	☐ 0. Walking
		☐ 1. Taxi
		☐ 2. Public transport (matatu)
		☐ 3. Motorbike
		☐ 96. Other → please specify ______________
17	How much did it cost to get to clinic today?	Local currency:
18	How long did it take you to get here today? (in minutes)	|__|__|__|
19	Are you registered with NHIF?	☐ 0. No

		☐ 1. Yes
20	Of your children under 18 years of age, how many are registered with NHIF?	|___|___|

Interview Notes:
How did the interview go today?
